# Generation of a canine anti-canine CD20 antibody for canine lymphoma treatment

**DOI:** 10.1038/s41598-020-68470-9

**Published:** 2020-07-10

**Authors:** Takuya Mizuno, Yukinari Kato, Mika K. Kaneko, Yusuke Sakai, Toshinori Shiga, Masahiro Kato, Toshihiro Tsukui, Hirofumi Takemoto, Akio Tokimasa, Kenji Baba, Yuki Nemoto, Osamu Sakai, Masaya Igase

**Affiliations:** 1 Laboratory of Molecular Diagnostics and Therapeutics, The United Graduate School of Veterinary Medicine, Yamaguchi University, 1677-1 Yoshida, Yamaguchi, Yamaguchi Prefecture 753-8515 Japan; 2 Department of Antibody Drug Development, Tohoku University Graduate School of Medicine, Miyagi, Japan; 3 New Industry Creation Hatchery Center, Tohoku University, Miyagi, Japan; 4 Laboratory of Veterinary Pathology, Joint Faculty of Veterinary Medicine, Yamaguchi University, Yamaguchi, Japan; 5 Nippon Zenyaku Kogyo Co., Ltd., Koriyama, Fukushima Japan; 6 Laboratory of Veterinary Internal Medicine, Joint Faculty of Veterinary Medicine, Yamaguchi University, Yamaguchi, Japan

**Keywords:** Targeted therapies, Pharmaceutics

## Abstract

Lymphoma is the most common hematological cancer in dogs. Canine diffuse large B cell lymphoma shows a relatively good response to treatment with multi-agent cyclophosphamide, doxorubicin, vincristine, and prednisone (CHOP) chemotherapy; however, the 2-year survival rate is as low as 20%. For human B cell type lymphoma, the anti-CD20 chimeric antibody, rituximab, was developed two decades ago. The combination of rituximab and CHOP chemotherapy was highly successful in improving patient prognosis. However, no anti-canine CD20 antibody is available for the treatment of canine lymphoma. During this study, a rat anti-canine CD20 monoclonal antibody was established. We also generated a rat-canine chimeric antibody against canine CD20 designed for clinical application. This chimeric antibody (4E1-7-B) showed in vitro antibody-dependent cell-mediated cytotoxicity (ADCC) and complement-dependent cytotoxicity (CDC) against the canine B cell lymphoma cell line CLBL-1. Moreover, to obtain stronger ADCC activity, a defucosylated 4E1-7-B antibody (4E1-7-B_f) was also generated, and it showed tenfold stronger ADCC activity compared with 4E1-7-B. 4E1-7-B_f as well as 4E1-7-B suppressed the growth of CLBL-1 tumors in an immunodeficient xenotransplant mouse model. Finally, a single administration of 4E1-7-B_f induced considerable peripheral B cell depletion in healthy beagles. Thus, 4E1-7-B_f is a good antibody drug candidate for canine B cell type lymphoma.

## Introduction

Lymphoma is one of the most common hematological cancers in dogs, and is important as a point of comparison for human lymphoma^1^. Canine lymphomas are classified into T cell and B cell types according to their cell of origin. B cell lymphoma, the most common type which is similar to human diffused large B cell lymphoma (DLBCL), has a favorable prognosis compared to T cell lymphoma because the cyclophosphamide, doxorubicin, vincristine, and prednisone (CHOP) regimen of chemotherapy leads to remission in most cases. However, the 2-year survival rate is only 20%, and most cases recur after remission, finally proving refractory to chemotherapy^2^. Therefore, a novel therapy is required. Recently, several novel treatment strategies for canine lymphoma, including low molecular weight inhibitors, have been established^3^, leading to the expectation of an improved prognosis for dogs with lymphoma. Nevertheless, antibody treatment for canine lymphoma has not yet been established.

Among antibody therapies for cancer, rituximab, an anti-human CD20 antibody, is the oldest and most widely distributed. Rituximab is a chimeric antibody in which the constant region of the original anti-human CD20 mouse antibody was replaced with a human constant region^4^. Rituximab binds to human CD20 molecules and directly induces apoptotic cell death in addition to its function through antibody-dependent cell-mediated cytotoxicity (ADCC) and complement-dependent cytotoxicity (CDC)^5^. It has been used alongside multiple drug CHOP chemotherapy (R-CHOP) in the treatment of B cell type lymphoid tumors, including non-Hodgkin B cell lymphoma, with dramatic success^6,7^. Following the success of R-CHOP therapy, several kinds of CD20 antibody drugs were developed to obtain a more potent cytopathic effect on B cell lymphoma cells, and among them, ofatumumab and obinutuzumab were recently launched^8^. Obinutuzumab, in particular, is a glycoengineered CD20 antibody with reduced fucose modification of the heavy chain constant region^9^. Removal of fucose glycosylation from an antibody drug was expected to produce far greater ADCC activity^10^; in fact, a defucosylated anti-CCR4 humanized antibody drug was approved for the treatment of adult T cell leukemia^11^. Furthermore, CD20 is also expressed on normal mature B cells, so anti-CD20 antibody drugs are able to eliminate normal B cells. As a result, they have also been used for the treatment of autoimmune diseases, such as systemic lupus erythematosus (SLE), nephrotic syndrome, and Wegener syndrome^12^.

Human studies have suggested that anti-CD20 antibody therapy is an excellent candidate for the treatment of canine B cell lymphoma because, as is the case in humans, CD20 is expressed in both canine B cell lymphoma cells and canine normal mature B cells^13^. More than ten years ago, Impellizeri et al*.* reported that based on flow cytometry analysis, rituximab did not bind to canine CD20^14^. However, there is an anti-human antibody that cross-reacts with canine CD20 in immunohistochemistry but not flow cytometry, meaning that this antibody is not capable of binding to the naïve canine CD20 molecule^13^. Since then, many laboratories have attempted to develop monoclonal antibodies against canine CD20 in order to establish an antibody therapy for canine B cell lymphoma. Aratana Therapeutics Inc. (Leawood, KS, USA) launched a therapeutic anti-canine CD20 antibody (Blontuvetmab) in 2015 in the United States and showed its clinical efficacy against B cell lymphoma in dogs in a conference abstract; however, peer-reviewed data are not available. Ito et al. presented an anti-canine CD20 antibody (clone 6C8) and showed its induction of antibody-dependent cellular phagocytosis (ADCP) activity in canine B cells^15^. Jain et al*.* developed an anti-canine CD20 antibody that cross reacted with human CD20^16^. Rue et al*.* also developed an anti-canine CD20 antibody (clone 1E4) and generated a chimeric antibody for therapeutic use^17^. They observed the in vitro cytotoxicity of this antibody via CDC and a decrease in the number of peripheral B cells in vivo in healthy beagles; however, the clinical efficacy in dogs with canine B cell lymphoma remain unknown.

In this study, we took the novel approach of developing an anti-canine CD20 monoclonal antibody in rats as a host species. We show that this antibody induced cell death in canine B cell lymphoma cell lines. Moreover, we generated a rat-canine chimeric version of this antibody and verified its function in vitro and in vivo.

## Results

### Establishment of the anti-canine CD20 antibody

**Figure 1:**
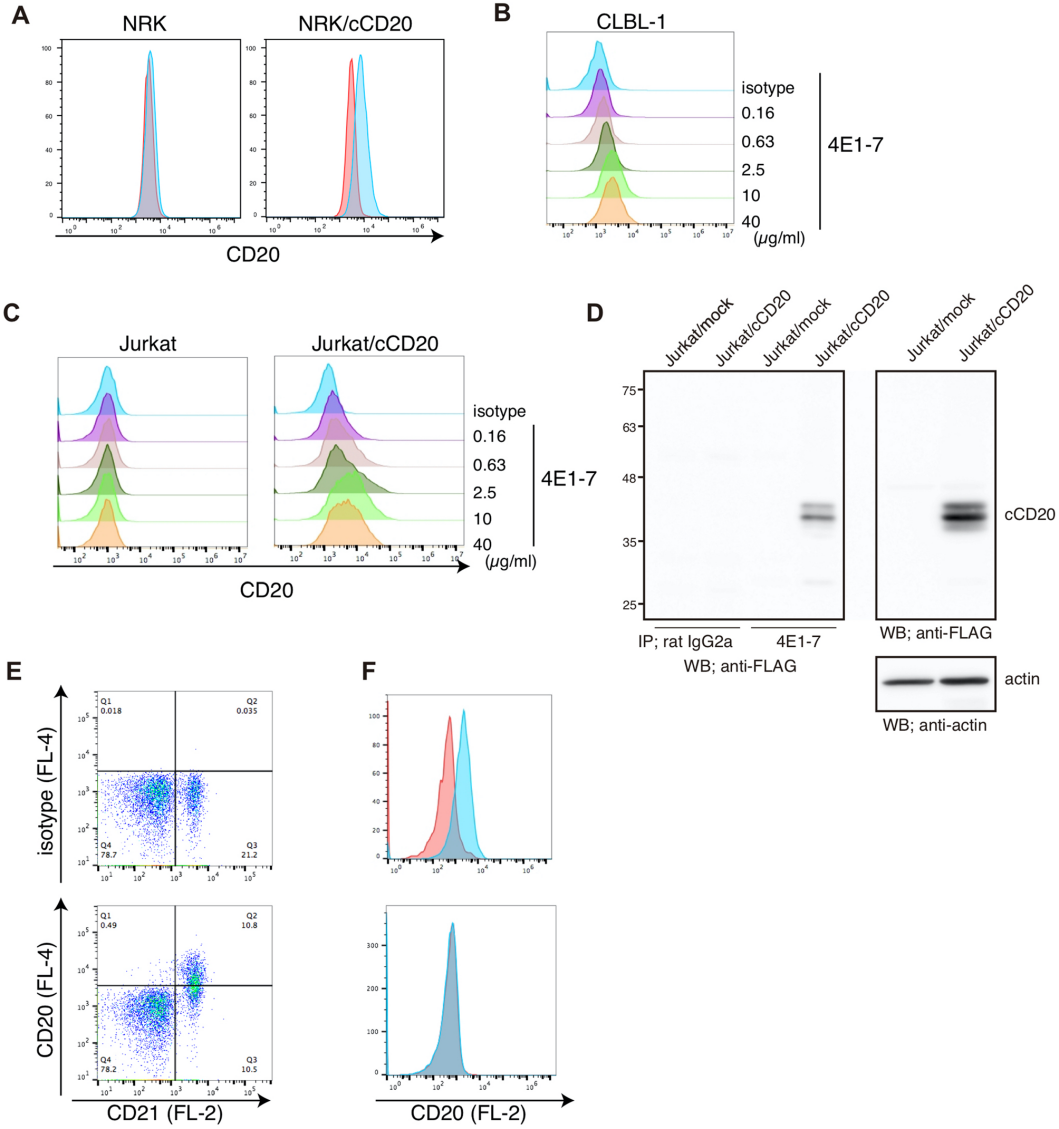
4E1-7 binds to canine CD20. (**A**) NRK and NRK/cCD20 cells were stained with 10 µg/ml of isotype control antibody (red) or anti-CD20 antibody, clone 4E1-7 (blue), followed by Dylight 649-labeled anti-rat IgG secondary antibody. (**B** and **C**) CLBL-1 (**B**) as well as Jurkat and Jurkat/cCD20 cells (**C**) were stained with the indicated amount of anti-CD20 antibody (4E1-7), followed by Dylight 649-labeled anti-rat IgG secondary antibody. (**D**) Cell lysates were extracted from each Jurkat and Jurkat/cCD20 cells and immunoprecipitated with 1 µg of anti-canine CD20 antibody (4E1-7) or rat IgG_2a_ antibody. Immunoprecipitated proteins (left) and whole cell lysate (right) were each separated by SDS-PAGE and transferred to the membrane, followed by western blotting with an anti-FLAG antibody. (**E**) PBMCs were isolated from four healthy beagles and stained with an anti-CD21-PE antibody and an anti-canine CD20 antibody (4E1-7), followed by Dylight 649-labeled anti-rat IgG secondary antibody. A representative image is shown. (**F**) Primary lymphoma cells were obtained from lymph nodes of two dogs with B cell lymphoma or one dog with T cell lymphoma and stained with anti-canine CD20 antibody (4E1-7) followed by an anti-rat IgG-PE secondary antibody. A representative image from the B cell lymphoma dogs is shown.

By immunization with NRK/cCD20 cells, an anti-canine CD20 monoclonal antibody (clone 4E1-7) was obtained, and its subclass was determined to be rat IgG_2a_ by flow cytometry. 4E1-7 reacted with NRK/cCD20 cells, but not parental NRK cells (Fig. 1A). The antibody also bound dose-dependently to the canine B cell lymphoma cell line CLBL-1 (Fig. 1B), but not to other canine lymphoma cell lines: GL-1, CL-1, Ema, UL-1, CLC, CLK, CLGL90, and 17–71 cell lines (data not shown). The antibody bound to the human lymphoma cell line Jurkat cells transduced to exogenously express canine CD20 (Jurkat/cCD20), but not to parental Jurkat cells (Fig. 1C). The anti-Flag antibody detected the bands of proper molecular weight (37 kDa) of canine CD20 in cell lysates from Jurkat/cCD20, but also in the 4E1-7 -immunoprecipitated cell lysates from Jurkat/cCD20 cells (Fig. 1D). However, the 4E1-7 antibody did not detect the bands in the cell lysates from Jurkat/cCD20 (data not shown), indicating that 4E1-7 recognized the nonlinear epitope. These results indicate that the 4E1-7 antibody is monoclonal and specific to the canine CD20 molecule.

### Anti-canine CD20 antibody binds to canine B cells and canine B cell lymphoma cells

To test the binding property of 4E1-7 to canine CD20 naturally expressed in canine B cells, canine PBMCs from 4 healthy beagle dogs were stained with 4E1-7 and anti-CD21 antibodies. Figure 1E showed that 4E1-7 bound specifically to the CD21^+^ population in canine PBMCs. We also assessed the binding property of this antibody to white blood cells, and neutrophils and monocytes were not stained by this antibody (data not shown). To prove that 4E1-7 also binds to B cell lymphoma cells from dogs, lymphoma cells were stained with 4E1-7 from 3 cases: two B cell lymphoma cases and one T cell lymphoma case (Fig. 1F). The B cell lymphoma cells from both cases were stained with 4E1-7, but those from the case with T cell lymphoma were not. This result shows that 4E1-7 also binds to canine CD20 naturally expressed in canine B cells.

### Anti-canine CD20 antibody has direct cell killing activity via apoptosis

**Figure 2:**
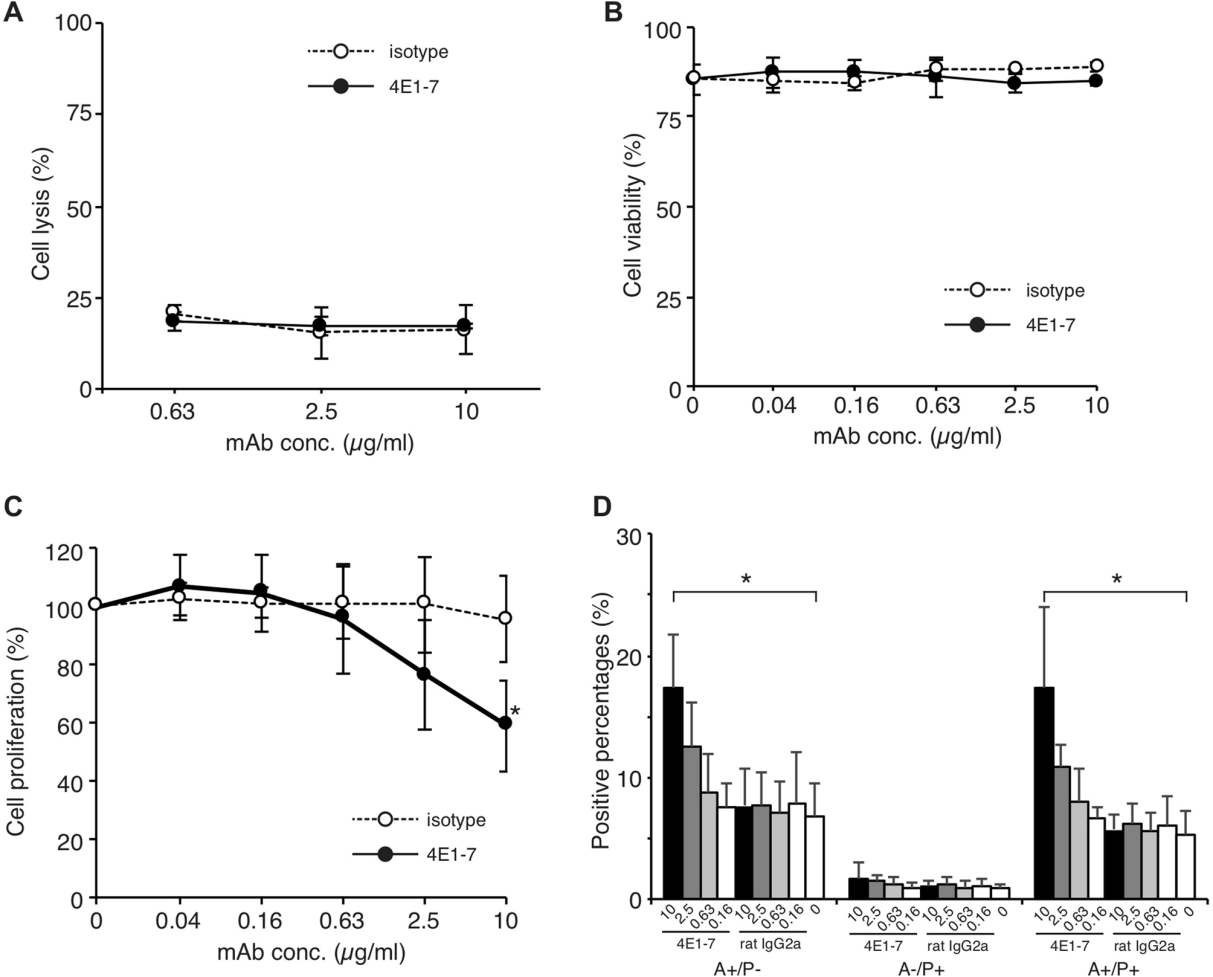
The 4E1-7 antibody induces cell death in CLBL-1 cells. (**A**) ADCC activity of 4E1-7 CLBL-1/luc cells were treated with the indicated amount of anti-CD20 antibody (4E1-7) or rat IgG_2a_ isotype control antibody for 20 min, followed by the addition of LAK cells. After 4 h of incubation, luciferase activity in living cells was measured using a luminometer after cell lysis. Mean ± SD of three independent experiments are shown. (**B**) CDC activity of 4E1-7 CLBL-1 cells were treated with the indicated amount of anti-CD20 antibody (4E1-7) or rat IgG_2a_ isotype control antibody for 15 min, followed by the addition of rabbit complement. After 90 min, viability was counted by trypan blue dye exclusion assay. Mean ± SD of three independent experiments are shown. (**C**) Direct cell killing activity of 4E1-7 CLBL-1 cells were incubated with the indicated amount of anti-CD20 antibody (4E1-7) or rat IgG_2a_ isotype control antibody for 72 h, followed by addition of the CCK-8 reagent. Cell proliferation rate was calculated. Mean ± SD of three independent experiments are shown. * Indicates P < 0.05. (**D**) Annexin V assay of 4E1-7 CLBL-1 cells were incubated with the indicated amount of anti-CD20 antibody (4E1-7) or rat IgG_2a_ isotype control antibody for 72 h, collected, and finally stained with annexin V and PI. The percentages of each annexin V positive and PI negative cells (A + /P-), annexin V negative and PI positive cells (A-/P +), annexin V and PI double positive cells (A + /P +), and annexin V and PI double negative cells (A-/P-) are shown. Mean ± SD of three independent experiments are shown. * Indicates P < 0.05.

To assess the functional activity of 4E1-7, several in vitro studies were conducted using CLBL-1 cell lines. To evaluate ADCC activity, CLBL-1 cells were treated with 4E1-7, followed by coculture with LAK cells for 4 h. Cell killing was not observed in this assay (Fig. 2A). Next, to examine CDC activity, CLBL-1 cells were treated with 4E1-7, followed by coculture with rabbit complement for 90 min. Again, cell killing was not observed in this assay (Fig. 2B). Finally, CLBL-1 cells were treated with 4E1-7 and incubated for 48 h. Cell proliferation assay showed that cell proliferation of CLBL-1 cells were suppressed dose-dependently by 4E1-7 (Fig. 2C). Annexin V staining of CLBL-1 cells treated with 4E1-7 showed that the annexin V^+^ propidium iodide (PI)^-^ population and annexin V^+^ PI^+^ populations increased with 10 µg/ml 4E1-7 treatment (Fig. 2D), indicating that the anti-proliferative effect of 4E1-7 in CLBL-1 cells was partly due to the induction of apoptotic cell death. These results suggested that 4E1-7 has an anti-proliferative function in CLBL-1 cells.

### Dog chimeric anti-canine CD20 antibody has strong ADCC activity

**Figure 3:**
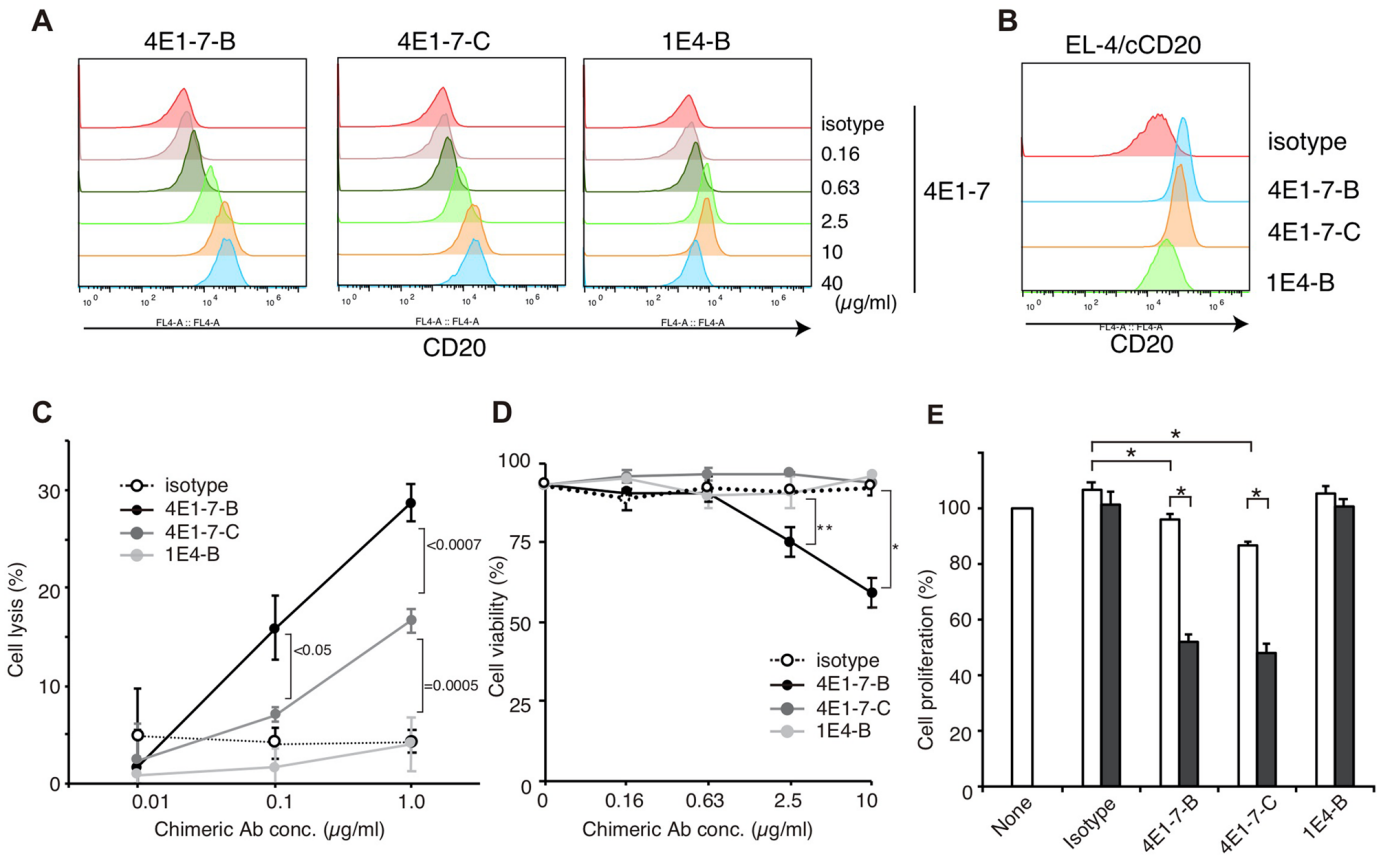
Chimeric antibody, 4E1-7-B, induces ADCC and CDC in CLBL-1 cells. (**A**) CLBL-1 cells were stained with the indicated amount of one of the anti-CD20 chimeric antibodies (4E1-7-B, 4E1-7-C, or 1E4-B), followed by Alexa 647-labeled anti-dog IgG secondary antibody. (**B**) EL-4/cCD20 cells were stained with 10 µg/ml of one of the anti-CD20 chimeric antibodies (4E1-7-B, 4E1-7-C, or 1E4-B), followed by Alexa 647-labeled anti-dog IgG secondary antibody. (**C**) ADCC activity of chimeric antibodies CLBL-1/luc cells were treated with the indicated amount of one of the anti-CD20 chimeric antibodies (4E1-7-B, 4E1-7-C, or 1E4-B) or the rat-dog IgG-B chimeric isotype control antibody for 20 min, followed by the addition of LAK cells. After 4 h of incubation, luciferase activity in living cells was measured using a luminometer after the cells were lysed. Three independent experiments were performed and means ± SD of cell cytotoxicity in triplicate from one representative result are shown. (**D**) CDC activity of chimeric antibodies CLBL-1/luc cells were treated with one of the anti-CD20 antibodies (4E1-7-B, 4E1-7-C, or 1E4-B) or the rat IgG_2a_ isotype control antibody for 15 min, followed by the addition of rabbit complement. After 90 min, luciferase activity in living cells was measured using a luminometer after the cells were lysed. Three independent experiments were performed and means ± SD are shown. * and ** indicate P < 0.0001 and P < 0.05, respectively. (**E**) Suppressive activity of cell proliferation of chimeric antibodies CLBL-1 cells were incubated with the indicated amount of one of the anti-CD20 antibodies (4E1-7-B, 4E1-7-C, or 1E4-B) or the rat IgG_2a_ isotype control antibody, for 15 min followed by treatment with 10 µg/ml of anti-dog IgG secondary antibody (black bars) for crosslinking, or mock control (empty bars). After 72 h of incubation, a CCK-8 reagent was added, and the cell proliferation rate was calculated. Three independent experiments were performed and means ± SD are shown. * indicates P < 0.05.

To determine the potential of the CD20 antibody as a therapeutic antibody for the treatment of canine B cell lymphoma, a rat-canine chimeric anti-canine CD20 antibody was prepared. As previously reported^18^, canine IgG has four classes, IgG-A, IgG-B, IgG-C, and IgG-D. Since research shows that IgG-B and IgG-C antibodies function via ADCC and CDC activity^19^, rat-dog chimeric anti-canine CD20 antibodies 4E1-7-B and 4E1-7-C were obtained. Previous research revealed that the anti-canine CD20 antibody (clone 1E4) had killing activity in CLBL-1 cells and the decreased number of peripheral B cells in healthy beagles^17^. Thus, we also prepared the chimeric antibody constructed from the variable region of 1E4 and from canine IgG-B for comparison. Flow cytometric analysis showed that both antibodies bound to CLBL-1 cell lines in a dose-dependent manner (Fig. 3A). These antibodies also bound to the EL-4/cCD20 cells, but Kd value of 1E4-B, 4E1-7-B and 4E1-7-C showed no difference (7.31 × 10^9^, 2.56 × 10^8^, and 3.83 × 10^8^, respectively) (Fig. 3B). We also confirmed the binding ability of 4E1-7-B to B cell lymphoma cells from 5 high grade B cell lymphoma cases (Supplementary Fig. 1).

Next, to establish the functional activity of the chimeric antibody, its ADCC and CDC activity as well as direct killing activity were examined. First, assessment of the ADCC mechanism showed, unexpectedly, that the 4E1-7-B and 4E1-7-C chimeric antibodies induced cell lysis of CLBL-1/luc cells in an antibody dose-dependent manner, and 4E1-7-B activity was significantly stronger than that of 4E1-7-C (Fig. 3C). The extent of cell lysis between the LAK cells varied in each dog, but the trends between the different dogs were similar. However, the 1E4-B antibody did not show any ADCC activity. Second, assessment of CDC by chimeric antibodies showed that in addition to 1E4-B and isotype control antibodies, 4E1-7-C induced no cell death in CLBL-1/luc cells. However, 4E1-7-B induced some cell death in a dose-dependent manner (Fig. 3D). Finally, the antibodies’ suppression of cell proliferation was assessed. Unexpectedly, treatment of CLBL-1 cells with 4E1-7-B and 4E1-7-C showed very weak suppression of cell proliferation compared with the parental 4E1-7 antibody (Fig. 3E). However, this effect was enhanced by crosslinking 4E1-7-B and 4E1-7-C antibodies with an anti-dog antibody. Conversely, 1E4-B showed no effects on cell proliferation, even in the presence of anti-dog IgG crosslinking.

### Defucosylated dog chimeric anti-canine CD20 antibody showed stronger ADCC activity

**Figure 4:**
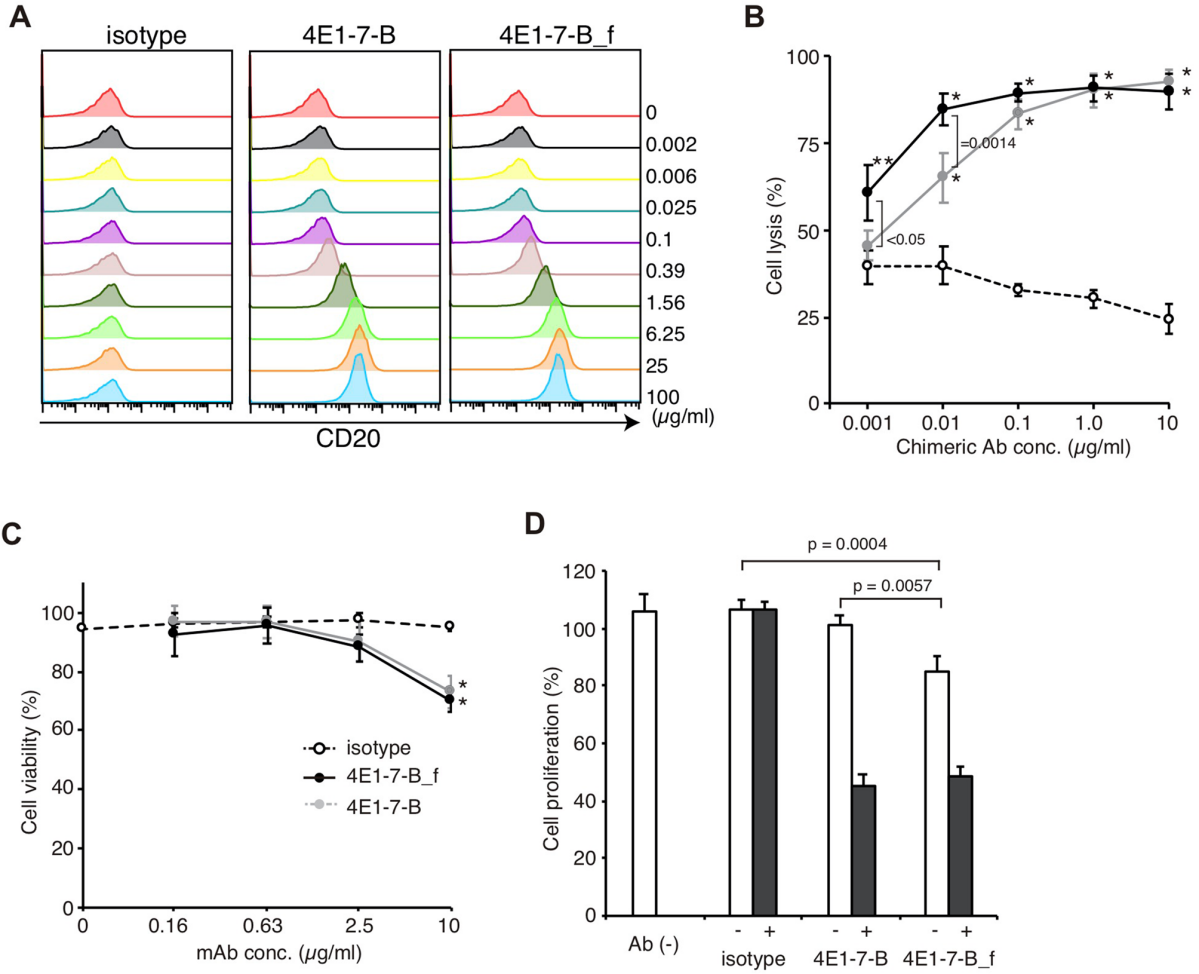
Defucosylated chimeric antibody, 4E1-7-B_f, induces more potent ADCC in CLBL-1 cells. (**A**) CLBL-1 cells were stained with the indicated amount of either of two anti-CD20 chimeric antibodies, 4E1-7-B or 4E1-7-B_f, followed by Alexa 647-labeled anti-dog IgG secondary antibody. (**B**) ADCC activity of defucosylated chimeric antibodies CLBL-1/luc cells were treated with the indicated amount of either of the two anti-CD20 chimeric antibodies (4E1-7-B or 4E1-7-B_f) or the rat-dog IgG-B chimeric isotype control antibody for 20 min, followed by the addition of LAK cells. After 4 h of incubation, luciferase activity in living cells was measured using a luminometer after the cells were lysed. Three independent experiments were performed, and the means ± SD of cell cytotoxicity in triplicate from a representative result are shown. * and ** indicate P < 0.0001 and P < 0.0004, respectively, compared to the isotype control. (**C**) CDC activity of defucosylated chimeric antibodies CLBL-1/luc cells were treated with either of the two anti-CD20 antibodies (4E1-7-B or 4E1-7-B_f) or the rat IgG_2a_ isotype control antibody for 15 min, followed by the addition of rabbit complement. After 90 min, luciferase activity in living cells was measured using a luminometer after the cells were lysed. Three independent experiments were performed and means ± SD are shown. * Indicates P < 0.0001. (**D**) Suppressive activity of cell proliferation of defucosylated chimeric antibodies CLBL-1 cells were incubated with the indicated amount of either of two anti-CD20 antibodies (4E1-7-B or 4E1-7-B_f) or the rat IgG_2a_ isotype control antibody for 15 min, followed by treatment with 10 µg/ml of anti-dog IgG antibody (black bars) for crosslinking, or a mock control (empty bars). After 72 h of incubation, a CCK-8 reagent was added, and cell proliferation rate was calculated. Three independent experiments were performed and means ± SD are shown.

To obtain stronger ADCC activity, the defucosylated chimeric antibody was generated using the cell lines deficient in FUT8^20,21^. The defucosylated antibody (4E1-7-B_f) bound to CLBL-1 cells in a similar manner to the 4E1-7-B antibody by showing the similar Kd value (2.41 × 10^8^) (Fig. 4A). Assessment of ADCC activity (Fig. 4B) showed that both 4E1-7-B and 4E1-7-B_f antibodies induced cytopathic effects in CLBL-1/luc cells, but 4E1-7-B_f showed almost 10 times greater cytotoxicity than 4E1-7-B. Assessment of CDC activity showed that the antibodies had similar effects (Fig. 4C). 4E1-7-B_f also suppressed cell proliferation of CLBL-1 cells, and this was enhanced by the addition of anti-dog IgG (Fig. 4D). However, the suppression rate was slightly higher in 4E1-7-B_f-treated CLBL-1 cells.

### Chimeric anti-canine CD20 antibody showed an anti-tumor effect in xenotranplanted mice

**Figure 5:**
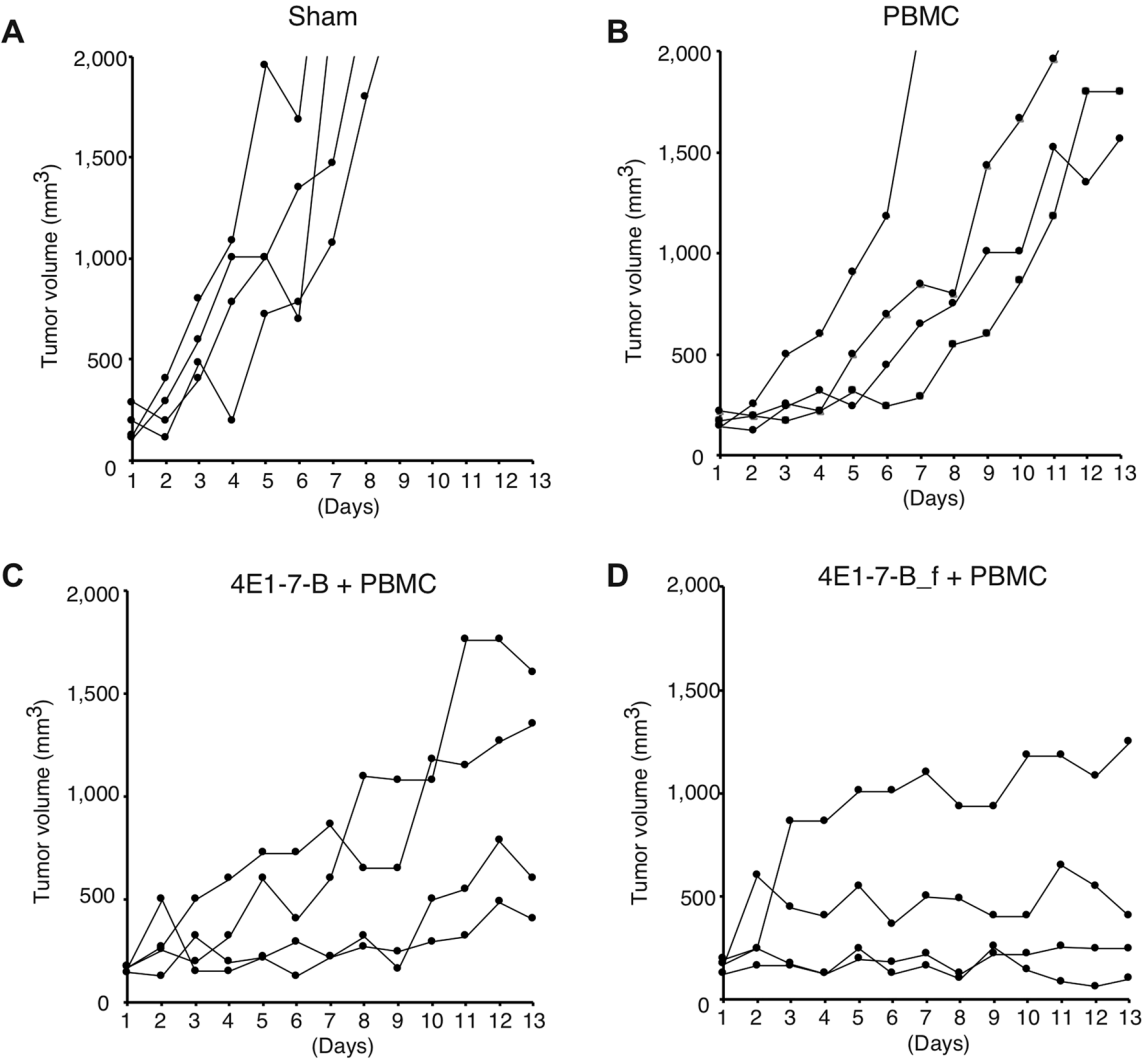
The defucosylated chimeric antibody, 4E1-7-B_f, suppresses tumor growth in NOD/SCID mice. (**A**–**D**) NOD/SCID mice were subcutaneously injected with CLBL-1 cells. After tumor size reached 150–250 mm^3^, mice were randomized into four groups to receive intraperitoneal treatment with either dog IgG (**A**), dog IgG (**B**), 4E1-7-B (**C**), or 4E1-7-B_f (**D**), as well as PBMCs around the tumor (**B**–**D**) every other day. Mice were euthanized as humane endpoints were reached or after 13 days of treatment.

To assess the anti-tumor effect of these chimeric antibodies in vivo, CLBL-1 cells were xenotransplanted into NOD/SCID mice, which were then treated with the chimeric antibody plus PBMCs (Fig. 5). Sole treatment with PBMCs (Fig. 5B) showed a slight delay in tumor growth compared with PBS treatment (Fig. 5A). However, administration of 4E1-7-B plus PBMCs (Fig. 5C) delayed tumor growth in two of four mice, and administration of 4E1-7-B_f suppressed tumor growth in all four mice (Fig. 5D), however tumor sizes at endpoint did not show a statistical significance between 4E1-7-B and 4E1-7-B_f groups (Supplementary Fig. 2).

### Chimeric anti-canine CD20 antibody decreases peripheral B cells in healthy beagles

**Figure. 6:**
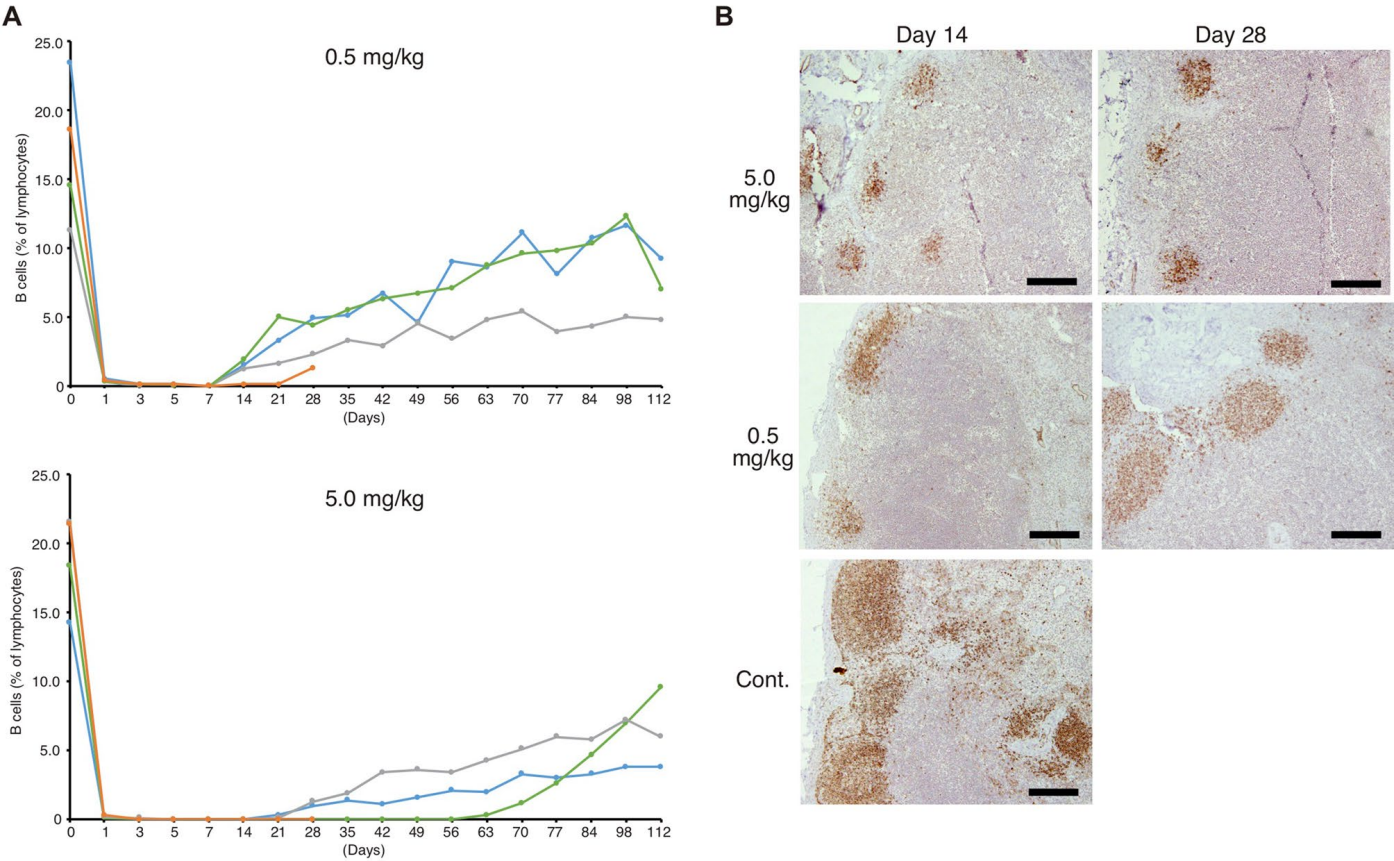
The defucosylated chimeric antibody, 4E1-7-B_f, depletes B cells in healthy beagle dogs. (**A**, **B**) Eight healthy beagles were intravenously inoculated with either 0.5 mg/kg or 5.0 mg/kg of the 4E1-7-B_f antibody (four dogs per dose) once on day 0. CD21^+^ B lymphocytes in peripheral blood were counted by flow cytometry every other day throughout day 1–7 then weekly after day 7. (**A**) In both groups, each line indicates a dog. One of the four dogs (orange line) from each group was euthanized on day 28 for analysis. (**B**) Lymph nodes were surgically obtained at days 14 and 28 from one dog from each group, and immunohistologically stained with an anti-CD79a antibody. Immunohistological staining of lymph node sample from a healthy beagle is shown as a control sample (Cont.) for comparison. Scale bar = 200 µm.

To prove that chimeric antibodies have the ability to lyse CD20^+^ B cells in dogs, two groups of four healthy beagles were injected intravenously once with chimeric antibodies (either 0.5 or 5.0 mg/kg), and then the percentages of peripheral CD21^+^ B cells were determined using flow cytometry (Fig. 6A and Supplementary Fig. 3). On the day after injection, the percentages and numbers of CD21^+^ B cells decreased to almost zero, and all dogs had no CD21^+^ B cells on day 3, which remained the case until day 7 in the 0.5 mg/kg group. On Day 14, one of the dogs of 0.5 mg/kg group still showed no CD21^+^ B cells, but the other three dogs showed a slight increase in the percentage of B cells. Compared with the 0.5 mg/kg dosage group, all four dogs in the 5.0 mg/kg dosage group showed no CD21^+^ B cells initially, but these cells gradually increased in two of the four dogs from day 21 onwards, one dog still had no CD21^+^ B cells on day 28 (when it was euthanized), and the remaining dog showed no CD21^+^ B cells until day 56. In both groups, the percentages of CD21^+^ B cells had not returned to the original percentages by day 119.

We also surgically removed the lymph nodes from one dog from each group on days 14 and 28. Immunohistochemical staining of B cells in the lymph nodes with the anti-CD79a antibody showed shrinkage of the positive area in the samples collected on day 14 and day 28 from the dog in the 5.0 mg/kg dose group and day 14 from the dog in the 0.5 mg/kg dose group, but the dogs in the 0.5 mg/kg dose group on day 28 showed no obvious effects on the CD79-positive area (Fig. 6B).

## Discussion

In this study, the hybridoma producing anti-canine CD20 antibody, clone 4E1-7, was established. This antibody suppressed cell growth of CD20-positive cell lines, partly due to apoptosis, but showed no ADCC and CDC activity. A few anti-canine CD20 antibodies, clone 6C8^15^, clone 1E4^17,22^, NCD1.2^16^ and Blontuvetmab (meeting abstract) have previously been described. Besides clone 1E4, none of these were shown to have an in vitro cytotoxic effect against canine CD20 positive cells. Clone 1E4, however, showed in vitro antibody-dependent cell phagocytosis (ADCP) activity and CDC activity^22^, and administration of a chimeric version of this antibody also induced peripheral B cell depletion in healthy beagles^17^. Unfortunately, the antibody cloned in our study, 4E1-7, did not exhibit ADCC and CDC activity, but did show direct cell killing activity. 4E1-7 turned out to be a rat IgG_2a_ subclass, and, generally, rat IgG_2a_ has been shown to exhibit ADCC and CDC activity^23^. The lack of ADCC activity of 4E1-7 may be attributable to the use of canine LAK cells as effector cells for the ADCC assay. Canine CD16 on LAK cells may not interact effectively with rat IgG_2a_. However, the reason for defective CDC activity remains unknown.

Interestingly, chimerization of the 4E1-7 antibody with canine IgG-B or IgG-C changed the characteristics of this antibody such that it gained potent ADCC and CDC activity but had reduced the suppressive effect on cell proliferation compared with the 4E1-7 antibody. We also compared these effects with the previously described 1E4 chimeric antibody (1E4-B)^17,22^, the only antibody shown to have in vivo B cell-reducing ability. 4E1-7-B had the highest ADCC activity followed by 4E1-7-C; meanwhile, 1E4-B showed no ADCC activity, even though it reportedly has ADCP activity^17,22^. This discrepancy might be because ADCC activity depends on CD16 expression on NK cells. On the other hand, ADCP activity depends on CD32 expression on macrophages. We observed CDC activity in 4E1-7-B-treated CLBL-1 cells, but not in 4E1-7-C or 1E4-B-treated cells. This was unexpected because previous research has shown that canine IgG-C has similar binding ability to human C1q as IgG-B^19^. The reason for this discrepancy is unknown; however, previous study only showed the binding ability to human C1q, but not CDC activity, and then canine IgG-B and IgG-C may have differential CDC activity, and the 4E1-7-C antibody was not strong enough to induce CDC in our system. Although 1E4-B displayed CDC activity during previous research^17,22^, no activity was detected in this study. We inferred that this results from differences in experimental conditions, such as incubation time; however, it was conclusively shown that 4E1-7-B displayed greater CDC activity than 1E4-B.

For therapeutic use of an antibody drug in the veterinary field, cost of the antibody is a pertinent issue, and so an antibody with high efficiency is desirable. For this reason, the defucosylated anti-canine CD20 antibody (4E1-7-B_f) was also generated and its activity compared with the 4E1-7-B antibody. As expected, CDC activity was not altered by defucosylation, but the 4E1-7-B_f antibody exhibited almost ten times greater ADCC activity than the 4E1-7-B antibody. We also observed slightly stronger suppressive effect on cell proliferation; however, the reason behind this discrepancy is not clear, although defucosylation may change the antibody structure and thereby influence the CDC activity.

To test the antibody in vivo, NOD/SCID mice were used for xenotransplantation of CLBL-1 cells. PBMCs were inoculated with an antibody to complement NK cell activity in the NOD/SCID mice^24^, in which NK activity is very low^25^ and rat NK cells also might not work efficiently with canine IgG. Treatment with PBMCs alone slowed tumor growth, which may be due to the small amount of NK activity in PBMCs. However, treatment with 4E1-7-B or 4E1-7-B_f and PBMC suppressed tumor growth. We did not show the statistical significance of the reduced tumor sizes at endpoints between two antibodies, which may be due to the small sample sizes, although 4E1-7-B_f tended to suppressthe tumor growth in four out of four mice.

Effects of the 4E1-7-B_f antibody on B cell depletion were impressive because even a single injection of 0.5 mg/kg induced transient disappearance of peripheral CD21^+^ B cells. Previously, the 1E4-B antibody was found to reduce peripheral CD21^+^ B cells after four sequential injections at a dosage of 10–30 mg/kg^17^, and this reduction was not to the levels that we observed in this study. Moreover, a single injection of 5.0 mg/kg 4E1-7-B_f induced a more sustainable decrease in peripheral CD21^+^ B cells with lymphoid depletion in the lymph nodes according to analysis of biopsies. We did not compare the efficacy of 4E1-7-B with 4E1-7-B_f in this study; however, the defucosylated antibody gave a remarkably strong effect of B cell depletion. In addition, despite the preliminary safety study using healthy beagle dogs, throughout this study, we had not seen any adverse events, such as general conditions, physical examination, complete blood counts except the slight and transient decline of platelet counts on the next day of treatment (supplementary Fig. 4), and serum biochemistry (data not shown).

In conclusion, we generated an anti-canine CD20 chimeric antibody, which had a high cytopathic effect on a CD20^+^ canine cell line. We also showed that defucosylation provided additive ADCC activity. This antibody also decreased the B cells efficiently in healthy dogs. Clinical trials for canine patients with B cell lymphoma using this antibody are now being prepared. We anticipate that this will become a treatment option and a candidate for a new therapeutic approach in the near future.

## Methods

### Cell culture

Canine B cell lymphoma cell line, CLBL-1^26^ (kindly provided by Dr. Barbara Rütgen from University of Veterinary Medicine Vienna) 17–71^27^ (kindly provided by Dr. Ilene D. Kurzman from University of Wisconsin) and GL-1^28^ (kindly provided by Dr. Munekazu Nakaichi from Yamaguchi University), canine T cell lymphoma cell lines: CL-1^29^ (kindly provided by Dr. Yasuyuki Momoi from The University of Tokyo), UL-1, Nody-1, Ema, CLK, and CLC^30^, CLGL-90^31^ (kindly provided by Dr. Maxey Wellman from The Ohio State University), , human lymphoblastoid cell line (Jurkat), mouse lymphoblastoid cell line (EL-4), and mouse myeloma cell line (P3U1) were all maintained in R10 medium, consisting of RPMI 1,640 supplemented with 10% FBS, 100 U/ml penicillin, 100 µg/ml streptomycin, and 55 µM β-mercaptoethanol. A rat kidney cell line (NRK), a human kidney cell line (HEK293T), and packaging cell lines (PLAT-E, PLAT-gp, and PG13) were cultured in D10 medium, consisting of DMEM with the same supplements as the R10 medium. These cell lines were cultured at 37 °C in a humidified incubator with 5% CO_2_.

Canine peripheral blood mononuclear cells (PBMCs) were isolated from healthy beagles that were kept as blood donors at the Yamaguchi University Animal Medical Center using lymphoprep (Axis-Shield Ltd., Dundee, Scotland) according to conventional methods. PBMCs were directly used for flow cytometry staining, as effector cells for inoculation of mice for tumor xenotransplant study, or were cultured in R10 medium containing 1,000 IU/ml of human recombinant IL-2 (Proleukin®, Chiron Therapeutics, Emeryville, CA) for 7 days prior to use in an ADCC assay as the lymphokine-activated killer (LAK) effector cells.

### Tumor cells from lymphoma cases

Primary lymphoma cells were obtained from enlarged lymph nodes from dogs that visited Yamaguchi University Animal Medical Center for diagnosis and treatment. Cytology was used to identify samples with greater than 90% of lymphoblasts. After diagnosis, the remaining sample cells were centrifuged and used for flow cytometry staining with owners’ consent.

### Molecular cloning of canine CD20 and canine IgG heavy chain and light chain constant regions

Canine CD20 and canine IgG heavy chain and light chain constant regions were molecularly cloned using stored cDNA from the cervical lymph node (for CD20) and spleen (for IgG) of a healthy beagle, as described in Doc S1.

### Establishment of stable cell lines

To obtain stable cell lines, the retroviral and lentiviral expression systems were used. The construction of expression vectors is described in Doc S2. Retroviral canine CD20 expressing vector (pMx-IP-cCD20-flag#4) with pCAGGS-VSVG, or lentiviral canine CD20 expressing vector (CSII-CMV-cCD20-Flag-IP#4) with pCVSVG and p8.9QV were transfected into the packaging cell line, PLAT-gp or HEK293T. NRK, Jurkat, and EL-4 cells were infected with the produced virus and cultured in the presence of puromycin (10 µg/ml, 4 µg/ml, and 4 µg/ml, respectively) to obtain the stable transfectants, NRK/cCD20, Jurkat/cCD20 and EL-4/cCD20, respectively.

To obtain a luciferase gene-expressing CLBL-1 cell line, Retroviral luciferase gene expressing vector (pMx-luc-IP#9) was transfected into PLAT-E cells. The produced retrovirus was added to a PG13 packaging cell line (PG13/luc). Retrovirus produced by PG13 was further added to CLBL-1 cells, followed by selection in the presence of 0.5 µg/ml of puromycin to obtain CLBL-1/luc cells.

### Generation of a monoclonal antibody

Hybridoma producing the monoclonal antibody against canine CD20 was obtained by immunizing the rat with NRK/cCD20 cells, according to our published protocol^32^, in accordance with the Yamaguchi University Animal Care and Use guidelines. Supernatant from the resultant hybridoma was collected and screened for reactivity to NRK/cCD20 and Jurkat/cCD20 using flow cytometry. Finally, a single hybridoma clone (4E1-7) was isolated using the limiting dilution method, followed by the adaptation of culture media to serum free media, Hybridoma SFM (Thermo Fisher Scientific Inc.). The supernatant was pooled, and the monoclonal antibody was purified using a HiTrap Protein A HP column (GE Healthcare UK Ltd, Buckinghamshire, England). Finally, the subclass of the antibody was determined using flow cytometry.

### Production and purification of recombinant antibody

To obtain the recombinant antibodies (4E1-7-B, 4E1-7-C, and 1E4-B), expression plasmids for the heavy chain and light chain of each chimeric antibody (Doc S3) were transfected into 293 cells using the Expi293 expression system (Thermo Fisher Scientific Inc.). The collected supernatant was purified using a HiTrap Protein A HP column (GE Healthcare) and desalted using a PD10 column (GE Healthcare). For production of the defucosylated antibody, BINDS-09 (core-fucose KO ExpiCHO-S) cells (https://www.med-tohoku-antibody.com/topics/001_paper_cell.htm) were used instead of HEK293T cells (29).

### Flow cytometry

Each cell was collected and washed with flow cytometry buffer (PBS with 2% FBS and 0.1% NaN3). 2 × 10^5^ cells were stained with the primary antibody: an anti-canine CD20 antibody (4E1-7, 4E1-7-B, 4E1-7-C, or 1E-4-B), a PE-labeled anti-CD21 antibody (Bio-Rad Laboratories, Inc., Berkeley, CA), or an isotype control (rat IgG_2a_, BioLegend, San Diego, CA, USA), followed by a secondary antibody: PE-labeled anti-rat IgG (SouthernBiotech, Birmingham, AL), Dylight 649-labeled anti-rat IgG (BioLegend), or Alexa 647-labeled anti-dog IgG (Jackson ImmunoResearch, West Grove, PA, USA).

To determine the subclass of 4E1-7, NRK/cCD20 cells were first stained with the anti-canine CD20 antibody (4E1-7), and then stained with either biotin-labeled anti-rat IgG-κ, anti-rat IgG-λ, anti-rat IgG1, anti-rat IgG_2a_, or anti-rat IgG_2b_ antibody, followed by incubation with streptavidin-PE (Thermo Fisher Scientific Inc.). Results obtained using a BD Accuri C5 flow cytometer (BD Biosciences) were analyzed using FlowJo v.10 software (Tree Star Inc., Ashland, OR, USA).

Binding affinity of each antibody was determined by flow cytometry. Each cell was stained by the serially diluted antibody, as described above. Kd value was determined using the Nonlinear Regression Michaelis–Menten curve fit by JMP14.0 software (JMP Japan, Tokyo, Japan).

### Western blotting and Immunoprecipitation

Cell lines were collected and stored at − 80 °C until use. SDS-PAGE and western blotting were done as described previously^33^. The anti-Flag antibody (clone M2, Sigma-Aldrich Crop., St. Louis, MO) and the anti-actin mouse monoclonal antibody (clone AC-15, Sigma-Aldrich Corp.) were used as primary antibodies. The horseradish peroxidase (HRP)-conjugated secondary anti-rat (Zymed, San Francisco, CA) and anti-mouse (Bio-rad Laboratories, Inc.) antibodies were used as secondary antibodies. The membrane was visualized by immersion in Western Lightning chemiluminescence reagent (Perkin Elmer, Foster City, CA, USA). Immunoreactive bands were visualized using the Luminescent Image Analyzer LAS 3,000 Mini instrument (FIJIFILM, Tokyo, Japan).

For immunoprecipitation, clarified cell lysates were pre-cleared with 10 µl of protein A/G agarose (Santa Cruz Biotechnology, Inc., Dallas, TX, USA) for 1 h at 4 °C with rotation. The resultant supernatant was mixed at 4 °C overnight with 1 µg of each antibody, which was premixed with 10 µl of protein A/G agarose for 1 h at 4 °C. The immunoprecipitates were washed three times with PBS and used for Western blotting as described above.

### Cell proliferation assay

CLBL-1 cells (2 × 10^4^ in 100 µl per well) were seeded into 96-well flat bottom microtiter plates containing each antibody in triplicate. In crosslinking experiments, CLBL-1 cells and antibodies were incubated for 15 min on ice, followed by the addition of 10 µg/ml of anti-dog IgG Fc-specific antibody (Jackson ImmunoResearch). After incubating for 72 h, 10 µl of CCK-8 solution (Dojindo, Kumamoto, Japan) was added to each well followed by incubation for a further 2 h. Absorbance at 450 nm was measured using an ARVO X4 microplate reader (Perkin Elmer).

### ADCC assay

ADCC assay was performed as previously described method^21^, using CLBL-1/luc cells (5 × 10^3^ in 100 µl per well), either rat IgG_2a_ (Thermo Fisher Scientific Inc.), 4E1-7, or chimeric antibodies, and IL-2-stimulated PBLs (an effector to target ratio of 20:1). Cell-mediated cytotoxicity was calculated as follows; triplicate wells were averaged, and percent lysis was calculated from the relative luminometer unit (RLU) data using the following equation: percent specific lysis = 100 × (spontaneous death RLU – test RLU)/(spontaneous death RLU – maximal killing RLU).

### CDC assay

CDC assay was performed as previously described method^21^, using CLBL-1/luc cells (5 × 10^5^ in 100 µl per well), either rat IgG_2a_ (Thermo Fisher Scientific Inc.), 4E1-7, or chimeric antibodies, and LOW-TOX®-H rabbit complement (Cedarlane, Burlington, Canada; final concentration 1:40). Live and dead cells were counted using trypan blue dye exclusion assay. For chimeric antibody experiments, CLBL-1/luc cells (1 × 10^5^ in 100 µl per well) were used instead of CLBL-1 cells, and live and dead cells were counted as described above.

### Apoptosis assay

CLBL-1 cells (1 × 10^5^ in 500 µl per well) were seeded into 24-well plates containing either rat IgG_2a_ or 4E1-7 antibodies. After 72 h of incubation, cells were stained using the FITC Annexin V Apoptosis Detection kit I (BD Biosciences) according to manufacturer instructions and were analyzed by flow cytometry.

### Mouse study

6–8 week-old NOD.CB17-*Prkdc*^*scid*^/J (NOD/SCID) mice were obtained from Charles River Laboratories Japan, Inc. (Kanagawa, Japan). Studies were conducted in a specific pathogen-free area in accordance with the Yamaguchi University Animal Care and Use guidelines. The CLBL-1 cells (1 × 10^6^ cells in 30 µl of PBS) were subcutaneously transplanted into the flank of NOD/SCID mice. After inoculation, the two perpendicular dimensions of the tumors were measured daily with calipers. Tumor volume was calculated from the measurements using the formula for the volume of an ellipsoid sphere [Tumor volume (mm^3^) = 1/2 × Length (mm) × Width^2^ (mm^2^)]. Once the tumors reached 150–250 mm^3^, the chimeric antibody (150 µg in 500 µl of PBS) was intraperitoneally injected every four days. Each time antibodies were injected, freshly isolated canine PBMCs (5 × 10^6^ cells each) from healthy beagles were suspended in 60 µl of PBS and injected into the flank surroundings of the tumors to complement the NK cell activity in tumors^24^ because NOD/SCID mice have low levels of NK activity^25^. After tumors reached 2,000 mm^3^, or 13 days has passed since the start of treatment, mice were sacrificed, and this is set as endpoints.

### Dog study

Eight healthy beagles (of four dogs each) were bred in house at the Zenoaq animal facility, and studies were conducted in a specific pathogen-free area and approved by the Animal Care and Use Committee of Zenoaq Nippon Zenyaku Kogyo Co., Ltd (Fukushima, Japan). One of two different dosages (either 0.5 mg/kg or 5.0 mg/kg) of defucosylated canine-rat chimeric anti-canine CD20 antibody (4E1-7-B_f) was intravenously injected once on day 0, forming the low and high dose groups, respectively. The percentages of CD21 + B cells in the peripheral blood were monitored at the indicated times using flow cytometry analysis (Animal Allergy Clinical LABORATORIES, Kanagawa, Japan). On day 14 and day 28, lymph nodes were obtained for immunohistopathological analysis from one dog from each the low and high dose groups under general anesthesia. Lymph nodes were sliced at 3 µm thickness and stained with an anti-CD79a antibody (clone HM57, dilution 1:50, Agilent Technologies Inc., Santa Clara, CA, USA) as previously described^34^.

### Statistical analysis

All statistical analyses were performed using JMP14.0 software (JMP Japan, Tokyo, Japan). Data were analyzed using one-way ANOVA followed by Tukey–Kramer multiple comparison tests. P < 0.05 was considered as significantly different.

## Supplementary information

Supplementary Information.
