# Supplementary material for: Generation of a canine anti-canine CD20 antibody for canine lymphoma treatment
Source: Sci Rep. 2020 Jul 10;10:11476. doi: 10.1038/s41598-020-68470-9 (PMC7351721; doi:10.1038/s41598-020-68470-9)
Supplement: Supplementary file 1 — Supplementary Information. [file 41598_2020_68470_MOESM1_ESM.pdf]

Generation of a canine anti-canine CD20 antibody for canine lymphoma treatment

Takuya Mizuno[1*], Yukinari Kato[2, 3], Mika K. Kaneko[2], Yusuke Sakai[4], Toshinori Shiga[5], Masahiro Kato[5], Toshihiro Tsukui[5], Hirofumi Takemoto[6], Akio Tokimasa[6], Kenji Baba[6], Yuki Nemoto[1], Osamu Sakai[1], Masaya Igase[1]

[1] Laboratory of Molecular Diagnostics and Therapeutics, The United Graduate School of Veterinary Medicine, Yamaguchi University, Yamaguchi, Japan.

[2] Department of Antibody Drug Development, Tohoku University Graduate School of Medicine, Miyagi, Japan

[3] New Industry Creation Hatchery Center, Tohoku University, Miyagi, Japan

[4] Laboratory of Veterinary Pathology, Joint Faculty of Veterinary Medicine, Yamaguchi University, Yamaguchi, Japan.

[5] Nippon Zenyaku Kogyo Co., Ltd., Koriyama, Fukushima, Japan

[6] Laboratory of Veterinary Internal Medicine, Joint Faculty of Veterinary Medicine, Yamaguchi University, Yamaguchi, Japan.

Molecular cloning of canine CD20 and canine IgG heavy chain and light chain constant regions Canine CD20 was amplified by PCR with YTM19 (5'-GCGCGGCCGCTCTCAGGAGTTCAGAGGGTGAG -3'; underlined: NotI) and YTM20 (5'-CAGAATTCTCAGGAAACAGGGGTGGATA -3'; underlined: EcoRI). These primers were synthesized based on the nucleotide sequences of canine CD20 (Accession number AB210085 registered in the NCBI database). Canine IgG heavy chain and canine IgG kappa light chain were amplified by PCR with YTM48 (5'-CCAGGTGACCCCATTCAGTGCTCAGGACAC -3'; named K9-IgG-5' in (19)) and YTM49 (5'-GGGTGGGGGGCTTGCTGGGTGCCGGGCG -3'; named IgG-REV4 in (19)), and YTM148 (5'-CACTGTCCGTGTCTGTCAGC -3') and YTM149 (5'- CCAAGGCCTGAGCTAGGAG -3'), respectively. The PCR reaction was conducted with KOD DNA polymerase -Plus- (TOYOBO, Tokyo, Japan) as per manufacturer instructions (denaturation at 95 °C for 2 min, 30 cycles of 95 °C for 30 sec and 56 °C for 30 sec, followed by 72 °C for 1-1.5 min). After the PCR reaction, 3' A overhangs were added by incubation at 72 °C for 10 min in the presence of Taq DNA polymerase. The amplified products were ligated into vectors using the TOPO TA Cloning kit (Thermo Fisher Scientific Inc., Waltham, MA, USA), and the plasmids was classified as pCR-cCD20, pCR-H-B, pCR-H-C, and pBS-κ. The nucleotide sequences of the amplified products of these plasmids were analyzed using an ABI PRISM3100-Avant sequencer (The DNA Core facility of the Center for Gene Research, Yamaguchi University).

Expression plasmids of canine CD20 and luciferase To tag the expression plasmid of canine CD20 with FLAG, pCR-cCD20 was amplified with primers YTM1233 (5'-ACGGATCCATGACAACACCCAGAAATTCAATG -3'; BamHI site underlined) and YTM1234 (5'- GTCGATGTCATGATCTTTATAATCAGGGATGCTGTCGT -3; FLAG tag underlined), followed by another PCR with YTM1233 and YTM838 (5'-TCACTACTTGTCATCGTCATCCTTGTAGTCGATGTCATG -3'; region overlapping YTM1234 underlined). The PCR product was cut with BamHI and ligated at the BamHI and SnaBI sites of pMXs-IP (kindly provided by Dr. Kurzman), resulting in pMx-IP-cCD20-flag#4. For the lentiviral expression plasmid, a BamHI-HincII fragment of pMx-IP-cCD20-flag#4 was inserted into the BamHI-HpaI site of CSII-CMV-MCS-IRES2-Bsd (provided by the DNA Bank, RIKEN BioResource Center, Ibaraki, Japan), resulting in CSII-CMV-cCD20-Flag-IP#4.

To generate the retroviral expression plasmid with the luciferase gene, the luciferase gene was amplified using primers YTM912 (5'- GCTAAGGTGGTGGACTTGGA -

3’) and YTM913 (5’- CCGCCCCGACTCTAGAATTA - 3’), cut with AgeI, and ligated into the HindIII-EcoRV sites of the pBluescript SK (-) plasmid with the HindIII-AgeI fragment of pGL4.50luc2 (Promega K.K., Tokyo, Japan). The resultant plasmid was called pBS-luc2. A XhoI-NotI fragment was cut out from pBS-luc2 and ligated into the XhoI-NotI sites of pMXs-IP (pMX-luc-IP#9).

Expression vectors of chimeric anti-canine CD20 antibody To obtain the variable regions of the heavy and light chains of the rat antibody, clone 4E1-7, 5’ rapid amplification of cDNA ends (RACE) was performed using a gene-specific primer according to Gilliland *et al*. (21). Total RNA was extracted from the hybridoma clone and reverse-transcribed using Superscript II (Thermo Fisher Scientific Inc.). The gene specific primers YTM171 (for the kappa light chain, 5’- TGCCATCAATCTTCCACTTGACA -3’; named rIgck-1 in (21)) and YTM172 (for the heavy chain, 5’- AAYTTTCTTGTCCACCTTGG -3’; named rIgG2ab-1 in (21)) for 5’ RACE. dCTP was added at the 5’ end of the resultant cDNA using terminal transferase (TOYOBO), followed by a PCR reaction using the primers YTM166 (5’- GGCCACGCGTCGACTAGTACGGGGGGGGGGGGGGG -3’; carrying poly-G at the 3’ end) and YTM171 (for the kappa chain) or YTM172 (for the heavy chain). The amplified product was further amplified using YTM166 and YTM173 (5’- GTTGTTCAWGARGCACACGACTGAGGCA -3’, named HBS-rck in (21)) or YTM174 (5’- AATAGCCCTTGACCAGGCAT -3’, named HBS-rG2a in (21)). The PCR product was ligated into a SmaI site of pBluescript SK (-), and the nucleotide sequences were determined using a sequence analyzer as described above. The variable region of the 4E1-7 kappa chain and the constant region of the canine kappa light chain (pBS-κ) were assembled by overlapping PCR and ligated into the pCAGGS-MCS vector (pCAGGS-4E1-7VκCκ#25). The variable region of the 4E1-7 heavy chain and the constant regions of canine IgG-B (pBS-H-B) and IgG-C (pBS-H-C) were assembled by overlapping PCR and ligated into the pCAGGS-MCS vector (pCAGGS-4E1-7VH-CHB#31 or pCAGGS-4E1-7VH-CHC#6, respectively). The sequences of the variable regions of the 4E1-7 heavy chain and light chain have already been published (https://www.j-platpat.inpit.go.jp/c1800/PU/JP-2019-026625/B1C814C4B5CBC0387F2A5B92EB836AC03023BD0960BCD18A1375406D746F4F6E/11/en).

To generate the previously published chimeric antibody (1E4-B) (17) to compare antibody function, the variable regions of the 1E4 heavy and light chains were synthesized (Genscript, Piscataway, NJ) as a single-chain variable fragment based on the patent information. A plasmid coding chimeric antibody, 1E4-B, was constructed by overlapping PCR of the 1E4 scFV plasmid and the canine κ light chain (pBS-κ) or the canine IgG-B plasmid (pBS-H-B).

Supplementary Figure 1

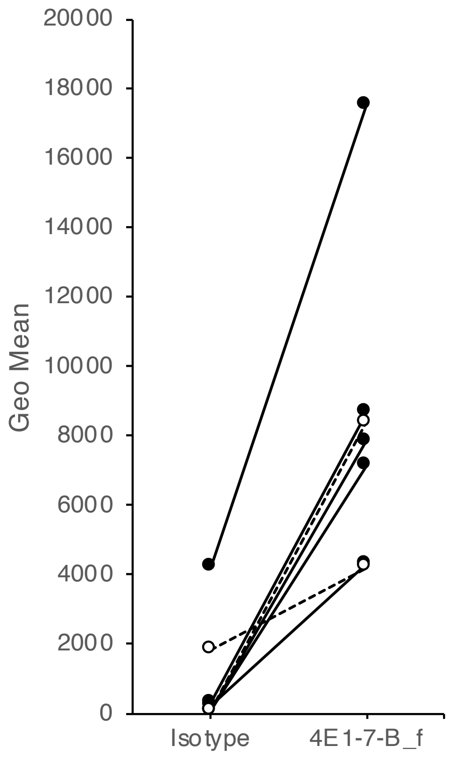

Supplementary Figure 1. 4E1-7-B antibody binds to primary B cell lymphoma cells. Primary lymphoma cells were obtained from lymph nodes of five dogs with B cell lymphoma, or primary peripheral CD21+ B cells were obtained from whole blood from two healthy dogs. Those were stained with chimeric anti-canine CD20 antibody (4E1-7-B) followed by an anti-dog IgG-Alexa 647 secondary antibody. Y-axis indicates the Geo Mean of fluorescent intensity stained by isotype control and 4E1-7-B. Each line represents each dog (Solid lines and dotted lines indicate lymphoma cells or primary B cells, respectively).

Supplementary Figure 2

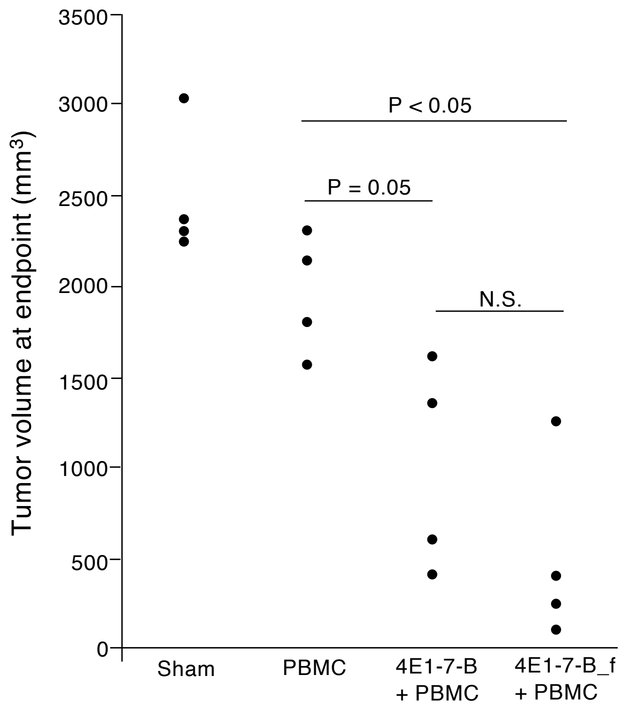

Supplementary Figure 2. Chimeric antibodies suppressed the tumor growth. Tumor volumes from each mouse (four mice from each group) at the endpoints were plotted. N.S.; not significant

Supplementary Figure 3

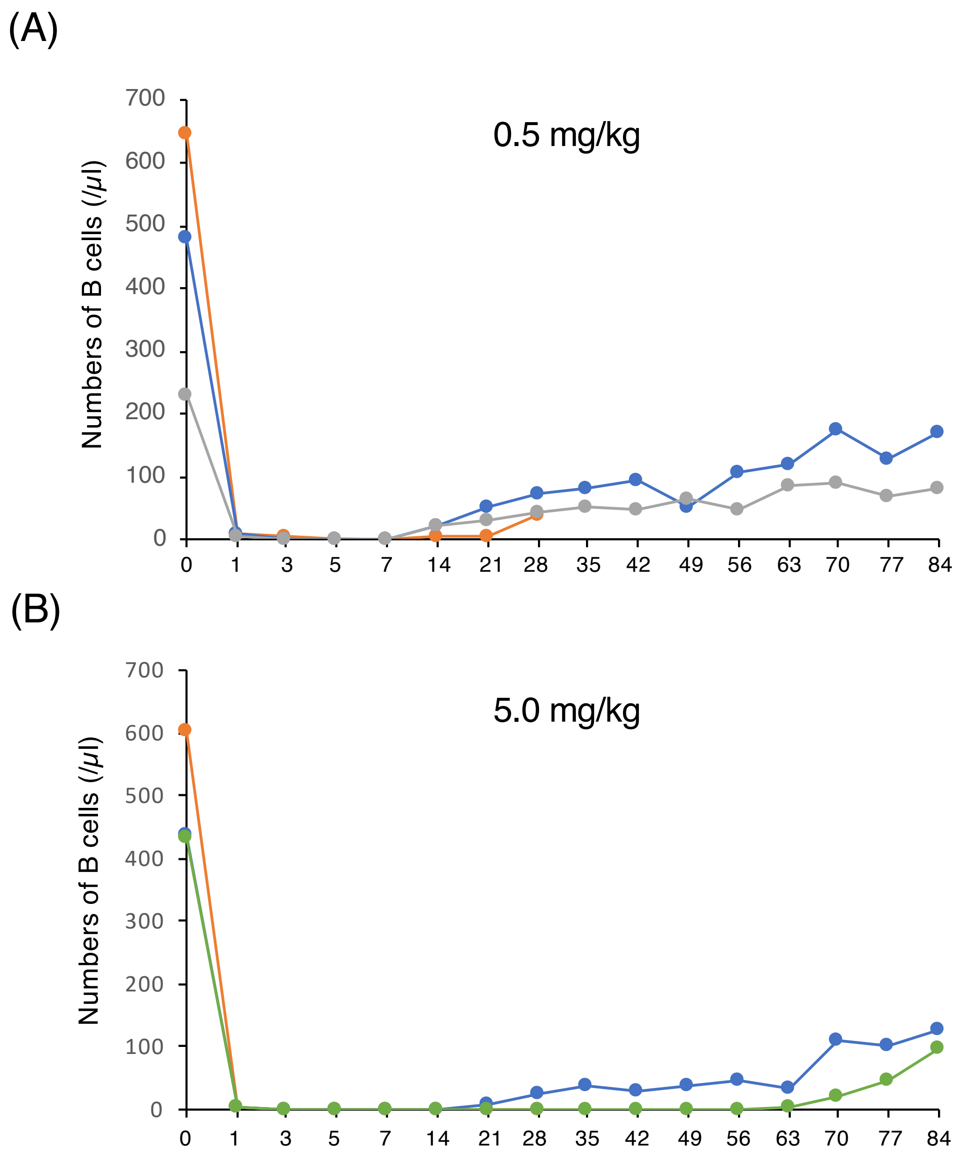

Supplementary Figure 3. The defucosylated chimeric antibody, 4E1-7-B_f, depletes B cells in healthy beagle dogs
(A, B) Eight healthy beagles were intravenously inoculated with either 0.5mg/kg or 5.0mg/kg of the 4E1-7-B_f antibody (four dogs per dose) once on day 0. Numbers of $CD21^{+}$ B lymphocytes in peripheral blood were calculated from the data of Figure 6. and lymphocytes count from CBC data. In both groups, each line indicates a dog. The color of each dog corresponds to that in Figure 6. One of the four dogs (orange line) from each group was euthanized on day 28 for analysis.

Supplementary Figure 4

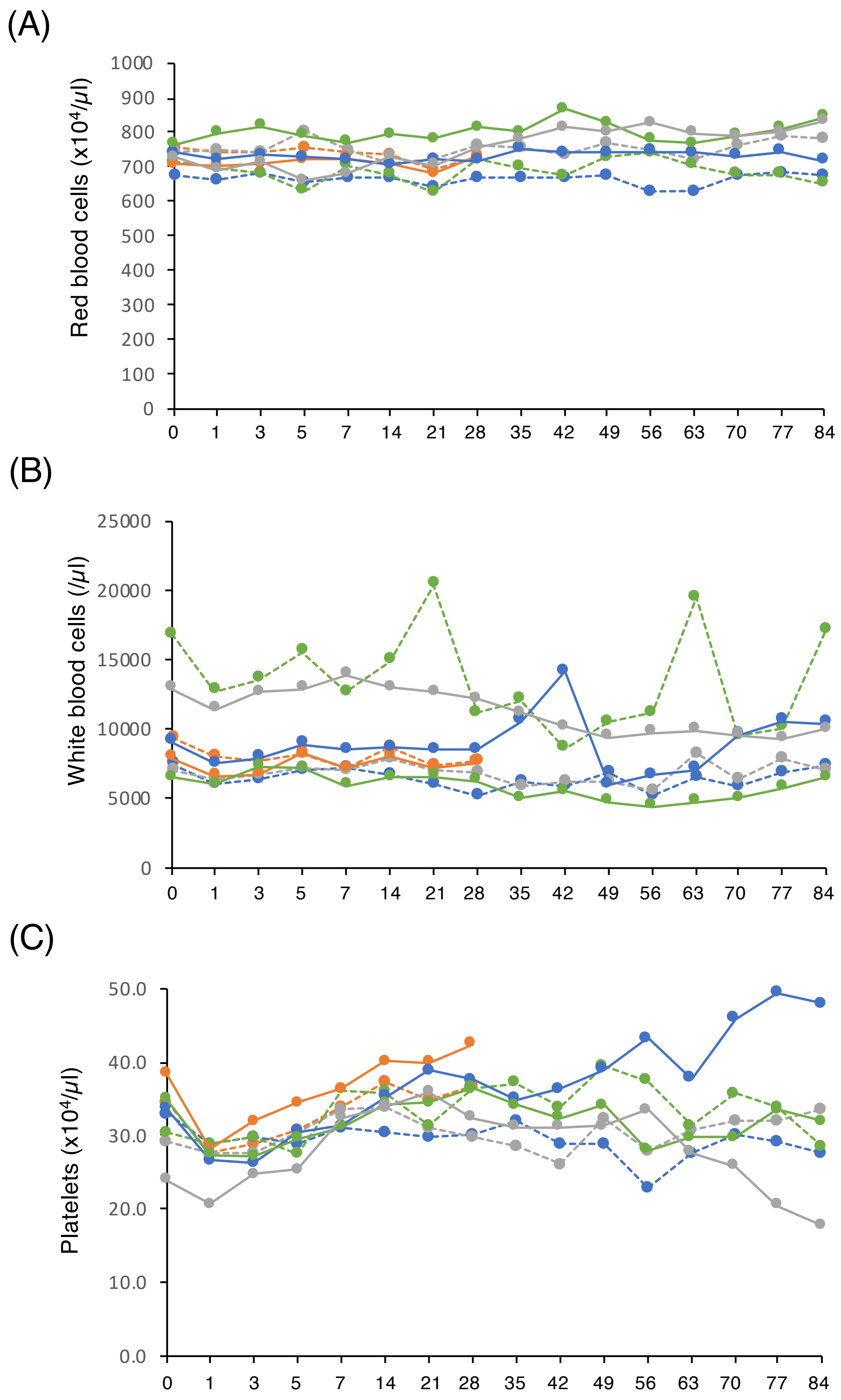

Supplementary Figure 4. The count of red blood cells (A), white blood cells (B), and platelets counts (C) in the dogs treated with the defucosylated chimeric antibody, 4E1-7-B_f. CBC data was obtained by XT-2000i (Sysmex corporation, Kobe, Japan) using EDTA-treated whole blood in the same course of experiment of Figure 6. Each line indicates a dog. The color of each dog corresponds to that in Figure 6, and dotted lines and solid lines indicated the dogs treated with 0.5mg/kg and 5.0mg/kg, respectively. One of the four dogs (orange line) from each group was euthanized on day 28 for analysis.

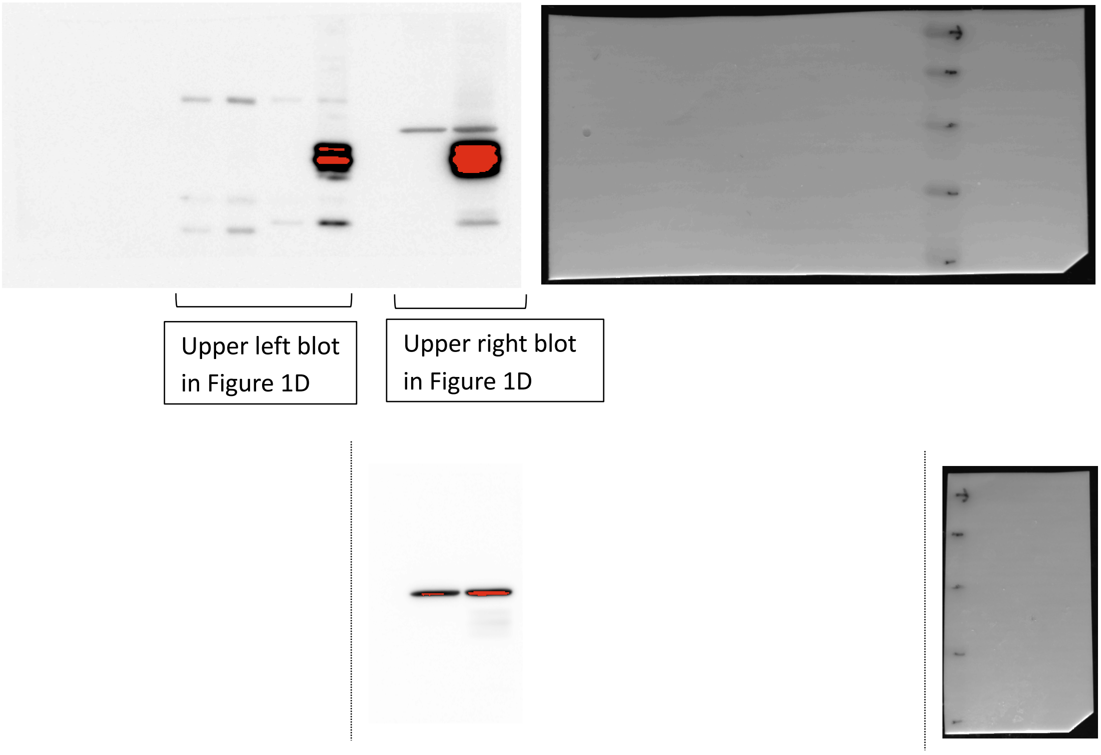

Supplementary Information 1. Full-length blots refer to Figure 1D. Upper left blot is the highly exposed blot of Figure 1D, and upper right blot is the same, but unexposed PVDF-membrane as marker is shown. The highly exposed blot is used here so that we can see the contour of membrane.

Lower blots are Actin blot in Figure 1D.

Dotted lines indicate the cut on the PVDF-membrane.
